# Allelic Selection of Amplicons in Glioblastoma Revealed by Combining Somatic and Germline Analysis

**DOI:** 10.1371/journal.pgen.1001086

**Published:** 2010-09-02

**Authors:** Thomas LaFramboise, Ninad Dewal, Katherine Wilkins, Itsik Pe'er, Matthew L. Freedman

**Affiliations:** 1 Department of Genetics, Case Western Reserve University School of Medicine, Cleveland, Ohio, United States of America; 2 Genomic Medicine Institute, Lerner Research Institute, Cleveland Clinic Foundation, Cleveland, Ohio, United States of America; 3 Department of Biomedical Informatics, Columbia University, New York, New York, United States of America; 4 Department of Computer Science, Columbia University, New York, New York, United States of America; 5 Department of Medical Oncology, Dana-Farber Cancer Institute, Boston, Massachusetts, United States of America; 6 Medical and Population Genetics Program, The Broad Institute of Harvard and Massachusetts Institute of Technology, Cambridge, Massachusetts, United States of America; Stanford University School of Medicine, United States of America

## Abstract

Cancer is a disease driven by a combination of inherited risk alleles coupled with the acquisition of somatic mutations, including amplification and deletion of genomic DNA. Potential relationships between the inherited and somatic aspects of the disease have only rarely been examined on a genome-wide level. Applying a novel integrative analysis of SNP and copy number measurements, we queried the tumor and normal-tissue genomes of 178 glioblastoma patients from the Cancer Genome Atlas project for preferentially amplified alleles, under the hypothesis that oncogenic germline variants will be selectively amplified in the tumor environment. Selected alleles are revealed by allelic imbalance in amplification across samples. This general approach is based on genetic principles and provides a method for identifying important tumor-related alleles. We find that SNP alleles that are most significantly overrepresented in amplicons tend to occur in genes involved with regulation of kinase and transferase activity, and many of these genes are known contributors to gliomagenesis. The analysis also implicates variants in synapse genes. By incorporating gene expression data, we demonstrate synergy between preferential allelic amplification and expression in *DOCK4* and *EGFR*. Our results support the notion that combining germline and tumor genetic data can identify regions relevant to cancer biology.

## Author Summary

Cancer is a disease of two distinct, but related, genomes: the inherited genome and the tumor genome. Despite the fact that the tumor genome arises from the germline, the genomes are typically studied as separate entities. For example, germline genetic studies focus on how inherited variation is related to a particular trait such as disease risk, whereas tumor genetic studies focus on areas of recurrent aberrations such as amplifications to identify genes involved in tumor biology. In this study, we integrated both germline and tumor genetic information to pinpoint areas of the human genome that are likely undergoing selection during the evolution of the tumor. Our results support the notion that combining germline and tumor genetic data can identify regions relevant to cancer biology.

## Introduction

Cancer is a disease of two related, but karyotypically distinct genomes: germline and somatic. Researchers typically focus on identifying genetic alterations by exclusively studying either the germline genome or the somatic genome. Germline genetic variants that play key roles in tumor biology (e.g., risk alleles) have typically been discovered using linkage and, more recently, association studies. On the other hand, somatic genetic elements important for tumor biology, such as amplifications, deletions, and point mutations, are usually identified by patterns of recurrence across tumor samples. Given the kinship between these two genomes, however, studies of cancer biology should be amenable to population genetic analysis, since the tumor cells can be considered descendants of a progenitor cell. In the population of tumor cells, lineages are subject to somatic versions of mutation, drift and selection [1]. We hypothesize that integrating germline allelic (i.e., genotypic) information with somatic amplification events could yield novel insights into the alleles that undergo selection during tumor evolution.

Associations between cancer risk alleles and somatic patterns are beginning to appear in the literature with increasing frequency. Preferential allelic amplification at candidate risk loci has been convincingly demonstrated in several mouse studies [2], [3] as well as in the analysis of the *AURKA* oncogene in humans [4], [5]. Additionally, a germline risk allele for colorectal cancer was demonstrated to be preferentially amplified (relative to the wild type allele) in tumors that were heterozygous for this single nucleotide polymorphism (SNP) [6]. More recently, a somatically acquired mutation in *JAK2* for myeloproliferative disorders was shown to arise preferentially on a particular haplotypic background [7]–[9]. These targeted studies of specific loci provide compelling evidence for the relationships between the germline and somatic genomes. One of the goals of our study is to perform a genome-wide query for such relationships.

**Figure 1:**
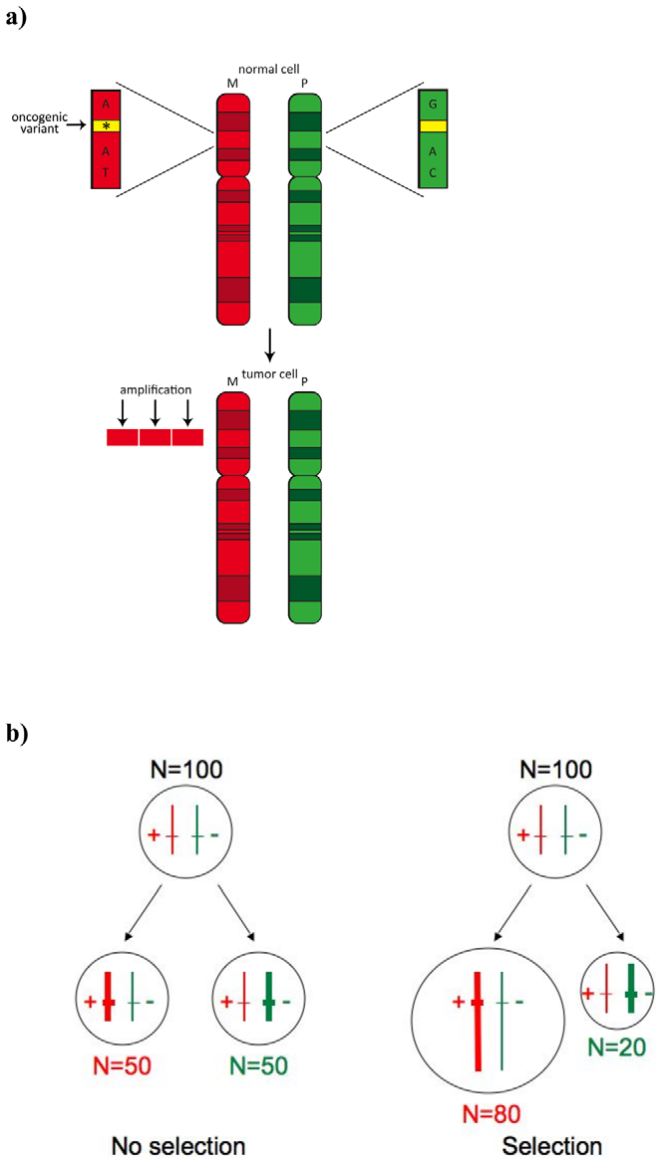
Biological rationale for selected allelic amplification in the tumor. (A) The individual inherits an oncogenic variant from the maternal chromosome M (top). This variant is not directly typed, but is captured via linkage disequilibrium by surrounding array SNPs (labeled with nucleotide residue). The paternal chromosome P harbors the wild-type allele. In the tumor environment (bottom panel), the oncogenic allele is activated via amplification, which confers a selective growth advantage to the cell. The amplified haplotype is detected from the SNP array data, and its preferentially amplified state is revealed through analysis of data from hundreds of patients. (B) In SNP array data, the underlying biological phenomenon will manifest itself in an abundance of amplicons harboring alleles that tag the inherited variant that provides a selective advantage when amplified. The ADT tests for over-transmission of a particular allele from heterozygous “parent” cells to the “affected” (amplified) homolog in the tumor cell and examines deviations from the null hypothesis of a 1∶1 transmission ratio.

We have developed a battery of statistical methods to query tumor DNA data for preferential allelic amplification [10]. These methods are designed to identify alleles that have likely been positively selected during tumor evolution within areas of copy number gain (Figure 1; Materials and Methods). One of these statistical tests, termed the amplification distortion test (ADT), is closely related to a well-known genetic test of association and linkage, the transmission disequilibrium test (TDT) [11]. Consider an example where *N*  = 100 tumors harbor an amplification and are heterozygous at a particular SNP locus (whose alleles are arbitrarily labeled *A* and *B*). Under the null hypothesis, on average 50 tumors will amplify one allele, and the other 50 will amplify the alternate allele since tumors typically amplify one of the two parental chromosomes [12]. In this sense, amplification is a somatic analog of Mendelian 50∶50 transmission in germline genetics. Significant deviations from a 1∶1 ratio (Figure 1B) are inconsistent with this null hypothesis and suggest that the particular allele – or a variant linked to that allele within the same amplified region – is advantageous to the tumor when amplified. Formally, we compare the observed number of germline heterozygotes amplifying the *A* allele to the Binomial(*N*, p = 0.5) distribution to obtain a two-sided P-value (Materials and Methods and [10]). Similar to the TDT, the ADT is robust to population stratification because the non-amplified homolog provides a perfectly matched control.

The recent National Cancer Institute-directed initiative, the Cancer Genome Atlas (TCGA; [13]), provides an ideal resource to test our hypothesis, furnishing SNP array data from multiple platforms across hundreds of glioblastoma multiformae tumors and matched normal samples. Since SNP arrays contain both the allelic and amplification information at hundreds of thousands of loci across the genome, the data are well-suited for our allelic distortion analysis. For each patient in the study, we made use of TCGA-generated SNP genotypes in the germline, as well as amplification status (generated on three separate probe hybridization-based platforms) and allelic amplification status for matched tumor DNA.

## Results

TCGA recently published (TCGA, 2008) the first report from a pilot study of over 200 human glioblastoma samples. As part of that study, the tumor DNA was interrogated at some 1.8 million loci using the Affymetrix SNP array 6.0, over 236,000 loci using the Agilent CGH microarray 244A, and at about 550,000 loci using the Illumina HumanHap550 array. We restricted our analysis to the 178 individuals for whom both germline and tumor array data from the Illumina platform were available. From these data, we extracted allelic copy number on a SNP-by-SNP basis; that is, for each individual, we inferred amplification status at each SNP locus, also identifying the amplified alleles in amplicon SNPs. As shown in Figure S1 (and observed in the TCGA manuscript), commonly (>5% of samples) amplified loci are restricted to several discrete – but wide – genomic regions. Such regions have a median length of 166 kb, and regions amplified in at least 15% of samples are usually even longer (median length 382 kb). Such broad regions of recurrent amplification can make it difficult to identify the target of these amplifications.

From a statistical standpoint, although we apply our test statistics across the genome, the ADT is not a genome wide test in the conventional sense because statistical power is expended only in a fraction of the genome. In practice, we only test loci with an amplification frequency sufficiently large to detect allelic selection. The power to detect selected allelic amplification of a SNP depends on its amplification frequency as well as its heterozygosity rate in the sample set [10]. For example, in the ADT, only SNPs with at least nine heterozygote calls in amplified samples have a chance of achieving a nominal two-tailed P-value <0.005. By deciding *a priori* that P-values above this level will not be considered significant in downstream analysis, we dramatically reduced the candidate loci under interrogation (Figure S2), decreasing the *de facto* number of SNPs to be tested by 91.9% from 547,458 to 44,132. This represents a far smaller multiple testing burden than in germline genome wide association studies (GWAS). Such reduction in testing burden improves our power to detect true effects.

**Figure 2:**
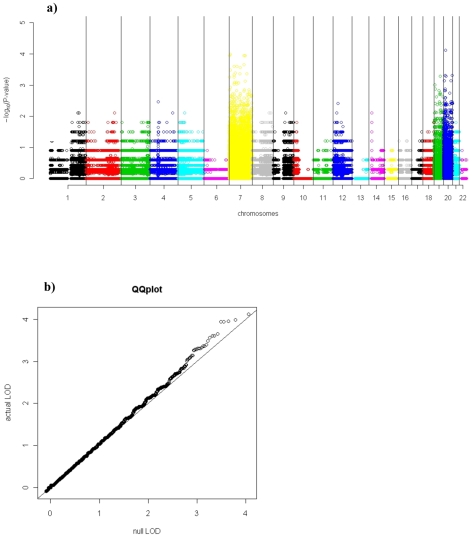
Genome wide ADT analysis of 178 TCGA glioblastoma samples. Manhattan-style plot (A) of amplification distortion P-value (y-axis, log_10_ scale) along the genome (x-axis). Save for the strongest hits, the ADT statistic follows the distribution expected under the null hypothesis, as demonstrated by the quantile-quantile plot (B) of P-values (log_10_ scale). Only SNPs with nine or more amplified heterozygous samples are presented, to avoid effects of discrete probabilities in a small sample.

Figure 2A presents the amplification distortion signals for SNPs tested along the genome. The statistical association for all but the top-scoring SNPs closely follows the distribution expected under the null hypothesis (Figure 2B), attesting to the validity of the assumptions. Although no single SNP achieves genome-wide significance, our results do yield a larger number of SNPs with lower P-values than would be expected by chance. Specifically, given the distribution of amplified heterozygotes in our data, we would expect an average of 114 SNPs to attain a P-value below 0.005 (95% confidence interval 98–132) under the null hypothesis of no random allelic amplification (Materials and Methods). In the actual data, 139 SNPs surpass this threshold (Table S1). This suggests that a subset of the SNPs among these top 139 is likely subject to selective allelic amplification. We checked the level of linkage disequilibrium (LD) these SNPs possess in HapMap CEU data (Table S1); 40 of these 139 SNPs are in strong LD in HapMap (*r^2^*≥0.7) with at least one other SNP within this set of 40 (Table S1, Figures S3, S4, S5). Note that our permutation scheme preserves LD structure, so that blocks of SNPs in LD can jointly contribute much of the signal not only in the actual data, but also during each permutation. Therefore, the 139 SNPs are still indeed more than expected by chance.

We should point out that one potential artifact arises from the fact that a germline copy number variant (CNV) gain might appear to be a somatic amplification when compared with the signal intensities from pooled normal samples. However, our methodology guards against this artifact in two ways (Materials and Methods). First, we call amplification in the tumor only if the intensity is greater than that of all normal samples in the study (Materials and Methods). Second, we call the amplified allele only if its allelic intensity is considerably larger in the tumor than in the matched normal. Finally note that, of our top 139 SNPs, only 23 (16.5%) are harbored in gains reported in the Database of Genomic Variants (DGV) (http://projects.tcag.ca/variation/), and of these only 8 are reported in more than three individuals in the database. Therefore, it is quite unlikely that germline CNVs significantly contribute to the ADT signal.

To investigate whether certain classes of genes may be driving our signals, we mapped each of the top 139 SNPs to the nearest gene (within at most 100 kb), which yielded 73 unique genes (Table S1). All but 22 of the 139 SNPs fell within 100 kb of a transcribed region, and 53 of the genes had single SNPs mapping to them. The largest number of significant SNPs mapping to the same gene was seven, all lying within the transcribed region of *NSPR1* (see Table S1 for *r^2^* LD values). We performed a gene ontology (GO) analysis [14] on the gene set to query for enrichment in specific annotations (Materials and Methods). The control set of genes for such analysis deserves special attention in this study, as gene sets may be over represented among our signals simply because they are over represented in amplified regions. To distinguish the signal driven by genes undergoing allelic selection from that driven by more general (non-allelic) amplification, this analysis was conducted by comparing our gene set with genes harboring (or near) SNPs that are recurrently amplified in our data. Thus, any observed enrichment in GO terms is above and beyond that which is due solely to general somatic amplification. This analysis allows us to query for signals from the allele-specific selection, controlling for those due to somatic amplification alone. The results are shown in Table 1 and Figure S6.

**Table 1:** Gene ontology analysis results.

GO term	P-value	q-value	Population Count	Study Count
positive regulation of protein kinase activity	9.03E-05	0.0471	30	6
phosphatase binding	3.20E-04	0.074	6	3
peptidyl-tyrosine phosphorylation	3.46E-04	0.074	14	4
synapse	6.24E-04	0.0851	42	6
positive regulation of catalytic activity	6.37E-04	0.0851	76	8

All terms showing enrichment (q-value <0.1) among genes identified by ADT analysis, as compared to the reference set of recurrently amplified genes, are shown.

Among our five significant (FDR q-value <0.1) GO enrichments is the cellular component term synapse (P = 0.0006). Of the 73 genes harboring (or very near) SNPs below the ADT threshold of P-value<0.005, six (*CADPS2*, *CHRM2*, *CHRNA4*, *GRM8*, *MAGI2*, and *SNAP25*) possess this annotation. Notably, the brain-related enrichment is independent of synapse related genes undergoing amplification in brain tumors, since general amplification is controlled for in this analysis. Therefore, the synapse annotation emerged strictly from the ADT selection signal among SNPs already in regions amplified in this brain-tissue tumor. This may be indicative of tumor selection for particular variants in these specific synapse-annotated genes.

**Figure 3:**
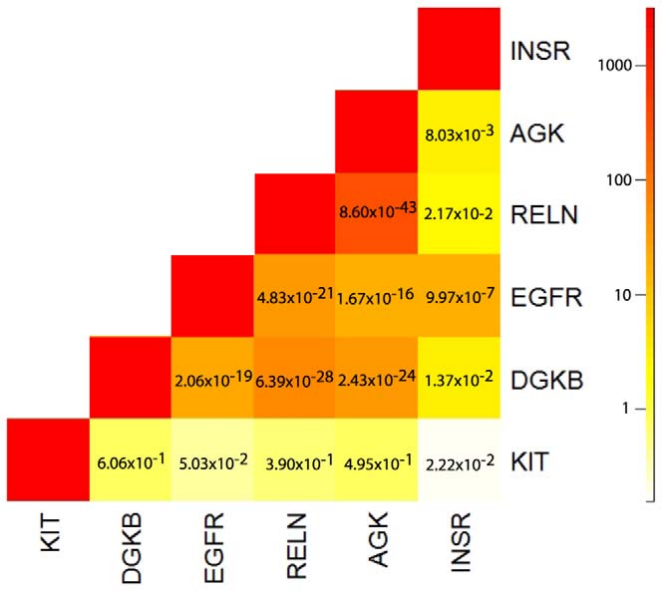
Heatmap of correlation, as measured by odds ratio estimates, between amplification status among six kinase/transferase activity genes showing signs of somatic allelic selection. Values above one indicate amplification correlation, below one anti-correlation. Fisher's exact P-values are given in each heatmap pixel.

Interestingly, the most significantly enriched GO terms (Table 1) were positive regulation of kinase activity (P = 9.03×10^−5^; Benjamini-Hochberg corrected q-value 0.0471) and positive regulation of transferase activity (P = 0.000132; Benjamini-Hochberg corrected q-value 0.0471). The six genes in our gene set associated with these GO terms are *AGK*, *DGKB*, *EGFR*, *INSR*, *KIT*, and *RELN*. Each of these genes is the closest to a single significant SNP, with the exceptions of *EGFR* with two such SNPs, and *RELN* with three such SNPs (see Table S1 for *r^2^* LD values). Furthermore (Table S1), each harbors one or more of the 139 SNPs within its transcribed region, with the exception of *DGKB* whose associated SNP is 66 kb downstream. To investigate the relative dependencies between the amplifications of these six genes, we examined the frequencies of their co-amplifications on a sample-by-sample basis. Of the six, four (*AGK*, *DGKB*, *EGFR*, and *RELN*) are located on chromosome 7. As expected, amplifications of these genes tend to co-occur far more often than would be expected by random assortment (Fisher's exact test P <10^−20^), largely due to the fact that amplicons often encompass most or all genes on the chromosome. The other two genes are located on chromosomes 4 (*KIT*) and 19 (*INSR*). Surprisingly, *INSR* is co-amplified with chromosome 7 genes in a statistically significant manner (Fisher's exact test P <10^−6^ for co-amplification with *EGFR*, odds ratio 14.2). On the other hand, *KIT* amplification is anti-correlated with that of the genes on chromosome 7 (P = 0.05 for anti-correlation with *EGFR*, odds ratio 0.5). Figure 3 provides an overview of the amplification association structure among these six genes. These correlation patterns may point to interdependent and/or alternative pathways that a tumor engages.

Given the preferential allelic amplification observed in some SNPs, we reasoned that an allele undergoing selection when amplified in a tumor may have an effect on disease risk as a germline predisposition variant. This principle has been previously demonstrated in mice [3] and in humans [6]–[9]. We therefore compared our list of the top 139 SNPs from our ADT analysis with the 406 SNPs reported in a recent GWAS for glioblastoma [15]. The rationale is that the variant that is selectively amplified in glioblastoma tumors may actually predispose the carrier to the initiation of the tumor, and thereby occur at a higher frequency in cases as compared to controls.

A pair of SNPs, rs4367471 and rs4132013 (*r* ^2^ = 0.78 in CEU HapMap population, Table S1) within a single haplotype block (in the European populations) in an intron of the *LHFPL3* gene, appears on both lists. Permutation analysis shows that an overlap of two or more SNPs between lists of these sizes (Materials and Methods) would be expected by chance only 2.1% of the time (P = 0.021). This is remarkable since the ADT makes use of no population control genotypes, while the GWAS study does not take tumor DNA into account. For both SNPs, the minor allele is overrepresented on amplified chromosomes (in the present study) and among glioblastoma cases (in the Wrensch et al GWAS). Among 24 amplified heterozygotes for rs4367471 in our study, 20 amplify the minor allele (P = 0.0015), while 27 of 32 amplified heterozygotes for rs4132013 amplify the minor allele (P = 0.00011). In the GWAS study, the rs4367471 minor allele frequency was 0.28 for glioblastoma cases, as compared to 0.23 for the disease-free controls (P = 0.00022). Similarly, the rs4132013 minor allele frequency was 0.24 for cases as compared to 0.19 for controls (P = 0.00042). The odds ratios were 1.28 for both SNPs after adjusting for population structure with the Eigenstrat software [16].

**Figure 4:**
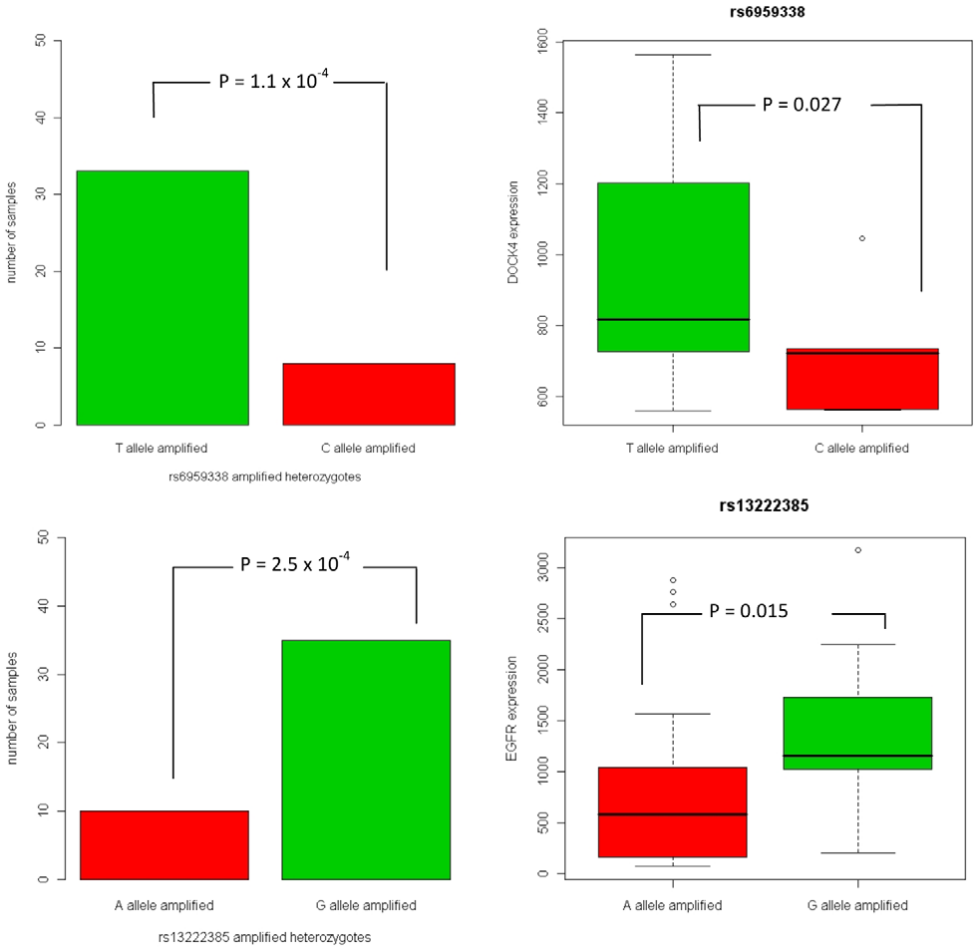
Selective allelic amplification and expression of SNPs in *DOCK4* and *EGFR*. The SNP rs6959338 in *DOCK4* shows preferential amplification of the T allele (upper left), as well as higher expression levels in samples amplifying T instead of C (upper right). Similarly, rs13222385 shows preferential allelic amplification (lower left) and expression (lower right).

The selective advantage gained by a cell amplifying a specific allele of a gene may be acting through direct changes in a gene product (e.g., a missense SNP) or by regulatory changes that modulate the quantity of gene product. The latter option is a testable hypothesis – it predicts that the amplification of the selected-for variant will be associated with elevated transcript expression levels. To investigate whether any of the detected signals of selected allele-specific amplification associates with expression, we integrated the expression data from the tumor samples with the genotype and amplification status. We considered Affymetrix U133A expression array data from the 154 individuals in our sample set for which the data was available from the TCGA website. Our list of top SNPs includes 65 whose nearest (as measured by base pair distance to transcribed region) gene is represented on the expression array. Of the 65 SNPs, only 28 had at least 5 examples of each SNP allele being amplified among the 154 samples, and were thus available for testing this association. Topping this list of 28 SNPs were rs6959338 and rs13222385, intronic variants in *DOCK4* (chr 7q31.1) and *EGFR* (chr 7p11.2), respectively: rs6959338 shows amplification of the T allele over the C allele in 33 of 41 amplified heterozygotes (P = 1.1×10^−4^); rs13222385 amplifies the G allele over the A allele in 35 of 45 amplified heterozygotes (P = 2.5×10^−4^). Intriguingly, the expression data shows statistically significantly higher expression in samples amplifying the selected-for allele than in those amplifying the other allele (Figure 4) in both *DOCK4* (P = 0.027) and *EGFR* (P = 0.015). To pursue this idea further, we tested the expression levels of the genes immediately flanking *EGFR* and *DOCK4* for association with amplification of the selected-for alleles. We were interested to discover that *LANCL2*, a gene 158 kb downstream from *EGFR*, has statistically significantly higher expression in rs13222385 heterozygotes amplifying the G allele than those amplifying the A allele (P = 0.0371). Taken together with the SNP's association with *EGFR* expression levels, this finding could point to a regulatory element, such as an enhancer for the allele or a linked variant.

## Discussion

We have presented a novel genome wide approach to identify genetic variants that are preferentially selected, via amplification, during tumor evolution. The ADT approach is statistically rigorous and is robust to the confounding effects of population stratification. The non-amplified chromosomal homolog provides the ideal matched control for the amplified homolog, as it comes from the same individual. Although no single SNP individually achieves genome-wide significance under the ADT (likely due to a lack of power owing to limited sample size [10]), our data does show enrichment in strong ADT signals as compared to chance. Currently, we are performing similar analyses in areas that have undergone copy number loss.

Our integrated analysis of genes harboring (or near) SNPs undergoing apparent allelic selection has revealed intriguing pathways and annotations. As revealed by the GO analysis, many of the variants showing ADT signals with P-values <0.005 are located within genes related to kinase activity. The fact that both the *EGFR* and *KIT* kinases reach statistical significance is of particular interest. *KIT* expression is often observed in gliomas, and imatinib (which is known to inhibit c-Kit) is currently being evaluated in clinical trials [17]–[21]. The correlation/anti-correlation relationships among these implicated genes may highlight glioblastomas that utilize different pathways and may therefore represent distinct subtypes of tumors that may be clinically relevant as has been recently described [22].

We also observed particular instances of the selectively amplified alleles driving higher expression in *DOCK4* and *EGFR*. *DOCK4* was originally isolated in a screen to identify homozygous genomic deletions during tumor progression in a mouse model and is part of a larger family of atypical guanine exchange factor (GEF) for Rho family GTPases [23]. Rho GTPases are highly conserved molecular regulators of cytoskeletal dynamics and influence many cellular processes including cell polarity and migration [24]. Interestingly, it has been previously shown that suppression of *DOCK4* RNA reduces dendritic growth and branching in hippocampal neurons, while overexpression enhances these processes [25]. Moreover, increases in Dock180 levels, another Dock family member, enhanced migratory and invasive capacity in vitro, while inhibition of expression significantly reduced glioma cell invasion in vitro [26]. Therefore, we speculate that *DOCK4* influences the invasive potential of gliomas and that the *DOCK4* alleles may differentially modulate this potential. The role of *EGFR* in glioma biology is well established [27] (and references therein). Somatically acquired mutations of *EGFR* are commonly (∼40%–50%) observed in gliomas, and the EGFR pathway is commonly targeted in this disease [13], [28]–[32]. Our results further substantiate the importance of *EGFR* and demonstrate that particular alleles play important roles in determining EGFR expression levels. It will be of interest to study if expression differences in this gene lead to amplified or diminished phenotypic consequences. Indeed, a recent article demonstrates that subtle alterations in expression levels can lead to dramatic phenotypic consequences [33].

The apparent selection of specific inherited alleles when amplified is consistent with several biological interpretations. The data can be considered in the context of Knudson's two-hit hypothesis [34] in that the associated SNP alleles are inherited variants (or capture variants via linkage disequilibrium) that provide a selective advantage when amplified. Indeed, it has been demonstrated that inherited alleles of a locus (e.g., the Arg72 and Pro72 variants of *TP53*) can have differential mechanistic effects (e.g., apoptotic potential) [35]. Another explanation is that *cis*- acting germline determinants influence the acquisition of somatic mutations, which are subsequently acted on by selection. Elegant experiments supporting this hypothesis in mice and humans have recently been published [7]–[9], [36]. Third, one may hypothesize that a *somatic* mutation provides a selective advantage only when amplified on a specific haplotypic background, or is selected against if the mutation arises on other allelic backgrounds; that is, only certain alleles will tolerate the somatic mutation.

Since selection implies function, the loci identified in this study are high-priority candidates for further investigation. The results may provide a way to rationally identify subtypes of cancers that are driven by distinct risk loci. If this is the case, then genome wide association studies for cancer risk may benefit from typing matched tumor DNA samples, in addition to germline DNA, and performing an integrative analysis. Alleles that do not affect risk predisposition may still yield important clues with respect to acquired tumor traits, such as angiogenesis, tissue invasiveness, evasion of apoptosis, etc. Functional studies, such as allele specific RNA interference for protein coding regions or somatic cell knock-in of alleles, may shed light on the mechanistic consequences of the alleles.

In summary, we demonstrate that integrating information from germline and tumor genomes can reveal aspects of tumor biology that are not readily identified by studying each genome in isolation.

## Materials and Methods

### Data sets

We obtained glioblastoma array data (GBM Publication Data Freeze) from the ftp site of TCGA. We utilized three different data types – germline genotypes, amplification status, and allelic imbalance – from various hybridization-based platforms, downloaded from the TCGA ftp site. First, germline SNP genotypes (Illumina platform) for all normal samples were obtained. Second, we accessed copy number segmentation data (from Affymetrix SNP array 6.0, Illumina HumanHap550, and Agilent CGH array 244A) for tumor samples, providing genomic regions for each individual that are inferred to have constant copy number along with the estimated “raw” (non-integer) copy number of that segment. Third, we obtained the raw allelic A and B signals for all samples (Illumina BAF measure), tumor and normal. This provides a raw measure of allelic imbalance, commonly termed the “B allele frequency” (BAF), defined as 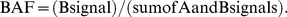



We also obtained Supplementary Table 7 from the Wrensch et al GWAS [15], which lists 406 SNPs with p<0.001 for association with high grade glioma comparing cases from San Francisco

Bay Area Adult Glioma Study, 1997–2006 (AGS) and the Cancer Genome Atlas (TCGA) to AGS and Illumina controls (iControls).

### Calling amplification

For each of the three platforms (Affymetrix, Agilent, and Illumina), we first inferred amplification at all 1.3 million autosomal SNPs represented by the Affymetrix and Illumina arrays combined, as follows. First, for each sample, all SNPs harbored in each genomic segment from the sample's copy number segmentation file (see above) are assigned that segment's raw copy number. A SNP is called amplified by the platform in a tumor sample if its raw copy number in that sample exceeds its raw copy number in all normal samples. This conservative amplification calling procedure accounts for local probe intensity effects, and avoids miscalling germline copy number variants as somatic amplifications. Note that this procedure, while conservative, is designed to include single-copy gains as well as high-level amplification events. Finally, for all downstream analyses, a sample is considered to harbor an amplification at a SNP if it is called amplified by at least two of the three platforms.

### Calling the amplified allele

For each SNP, we restrict the remainder of our analysis to individuals that are both heterozygous in the germline and amplified in the tumor at the SNP site. For each of these samples, we aim to determine which of the two alleles is amplified. Towards this end, we exploit the BAF measure described above. Since each sample is heterozygous in the germline, we expect the SNP's BAF measure to be near 0.5 in the germline. A tumor BAF larger than 0.5 is indicative of B allele amplification, and a BAF smaller than 0.5 is indicative of A allele amplification. However, bias in A and B intensity measures can result in deviations from these expectations. We therefore rely on the deltaBAF measure, defined as
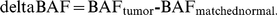



The expectation here is that A (respectively, B) allele amplification will result in a negative (respectively, positive) deltaBAF value. To avoid erroneous deltaBAF calls due to noisy probe intensities, we only have confidence in allele calls where |deltaBAF| >0.05. That is, for heterozygous (in the germline) samples that are amplified (in the tumor), we call A allele amplification if deltaBAF <−0.05 and B allele amplification if deltaBAF >0.05.

### ADT P-values

The procedure described above yields sample counts for A amplification and B amplification at each SNP. Let *n*_A_ and *n*_B_, respectively, denote these counts. Under the null hypothesis of random allelic amplification, *n*_A_ follows a Binomial(*n*_A_ + *n*_B_, 0.5) distribution. In other words, if there is no causal allele or site within an amplified region, the distortion signature of each SNP within the amplified region should conform to the null signature on the binomial distribution. Therefore, a (two-sided) P-value testing preferential allelic amplification may be performed by comparing *n*_A_ with this distribution in the obvious manner. The chance of a non-causal/non-associated allele within an amplified region being randomly selected enough times to result in a distortion (i.e. false positive) is α, where α represents a chosen level of significance as described in the following section.

### Permutation analysis and quantile-quantile (qq-)plot

Although not a genome-wide association scan, our approaches comprise many tests whose correlations are manifold and complicated. Furthermore, some regions harbor more amplifications than others and therefore have a higher *a priori* likelihood of displaying allelic distortion even under the null hypothesis. Therefore, analytically determining genome-wide significance from the test statistics is not straightforward. To address this, we developed a permutation procedure that assesses the significance of our results. For each run of the procedure, we first randomly determined – at each sample chromosome pair – whether to swap amplification status from the amplified allele to the non-amplified allele at all amplified (in the tumor) SNPs on the chromosome. This preserves haplotype and amplicon structure while destroying correlation between the two. We then recomputed the test statistics across the genome. In this manner, the amplification status of the samples is preserved, and we are randomly sampling from the null situation of non-preferential (random) amplification. For the ADT test, these simulations produced an average of 114 SNPs (95% confidence interval 98–132) surpassing the 0.005 threshold. For the qq-plot, the *q*th null P-value quantile was estimated by averaging the *q*th quantiles of P-values from 1000 permutations. Finally, the significance of the overlap between the ADT SNPs and the Wrensch et al GWAS SNPs was assessed by permuting, 1000 times, and retaining the 139 most significant SNPs for each permutation (since our actual data generated 139 top SNPs). These number of SNPs in each permutation that intersected with the Wrensch list was tallied for each permutation, which yielded the expected distribution expected by chance.

### GO analysis

For our GO analyses, we compared the gene list with a “gene universe” comprised of all genes that had any *a priori* chance of demonstrating preferential allelic amplification, at the P<0.005 nominal level, in our data. For a given gene, this depends upon many factors, including amplification frequency and allele frequencies of nearby array SNPs. We restricted the gene universe to genes that were within 100 kb of a HumanHap550 array SNP that is heterozygous and amplified in at least nine of our samples. This is reasonable, as these are the only genes (by definition) that have an *a priori* chance of having an associated SNP with ADT P-value below 0.005. This left 2696 genes as a reference set. Using the Ontologizer [37] software, we assessed our gene lists for enrichment in GO terms, as compared with this reference gene universe, using the Term-For-Term method and Benjamini-Hochberg correction.

### Gene co-amplification analysis

For each pair of genes, we constructed a 2×2 table of counts for number of samples in each category of amplification/non-amplification status for each gene. Using this table, we computed the odds ratio estimate for correlation between amplification of the genes, and assessed its significance using Fisher's exact test. For genes *X* and *Y*, this corresponds to the ratio of the odds of gene *X* being amplified in a sample with gene *Y* amplified to the odds of gene *X* being amplified in a sample without gene *Y* amplified.

### Incorporating expression

Expression levels from the Affymetrix 133A array were downloaded from the TCGA website. For each gene/SNP combination, expression differences between samples expressing each of the two alleles were computed using the non-parametric Wilcoxon rank sum test. The test was one-sided, since there was an *a priori* hypothesis that the preferentially amplified allele would result in a higher expression level.

## Supporting Information

Figure S1 Proportion of individuals (out of 178) amplified in the tumor, at each Illumina HumanHap550 SNP.
(1.01 MB TIF)

Figure S2 The number of individuals (out of 178) heterozygous in the germline and amplified in the tumor, at each Illumina HumanHap550 SNP.
(1.13 MB TIF)

Figure S3 (A) Chromosome 7: Condensed Haploview r2 LD Plot for the three ADT hits (rs10255873, rs10267828, rs1557841) in NXPH1. This condensed plot does not display SNPs in the region that are not ADT hits. The plot indicates that the SNPs are in LD in HapMap CEU data. (B) Chromosome 7: Condensed Haploview r2 LD Plot for the five ADT hits (rs6963353, rs40, rs2107479, rs1467344, rs10950366) in THSD7A. This condensed plot does not display SNPs in the region that are not ADT hits. The plot indicates that the latter two SNPs are in LD in HapMap CEU data. (C) Chromosome 7: Condensed Haploview r2 LD Plot for the seven ADT (rs2530552, rs425990, rs2530571, rs324389, rs10267134, rs10278663, rs17199888) in NPSR1. This condensed plot does not display SNPs in the region that are not ADT hits. The plot indicates that the first three SNPs are in reasonable LD in HapMap CEU data, and the same holds for the latter three SNPs. Any blank red blocks indicate r2  = 1.0 (100). (D) Chromosome 7: Condensed Haploview r2 LD Plot for the three ADT hits (rs6965611, rs2464946, rs11238181) in a region with no known gene. This condensed plot does not display SNPs in the region that are not ADT hits. The plot indicates that the first two SNPs exhibit some LD in the HapMap CEU data.
(1.09 MB TIF)

Figure S4 (A) Chromosome 7: Condensed Haploview r2 LD Plot for the three ADT hits (rs1963647, rs262375, rs264375) in RELN. This condensed plot does not display SNPs in the region that are not ADT hits. The plot indicates that the SNPs display some level of LD in HapMap CEU data. (B) Chromosome 7: Condensed Haploview r2 LD Plot for the four ADT hits (rs10273020, rs4730037, rs4367471, rs4132013) in LHFPL3. This condensed plot does not display SNPs in the region that are not ADT hits. The plot reveals that the first two SNPs are in strong LD in HapMap CEU data, as blank red blocks indicate r2  = 1.0 (100). The latter two SNPs also show LD in HapMap CEU data. (C) Chromosome 7: Condensed Haploview r2 LD Plot for the three ADT hits (rs9641684, rs2189601, rs9969220) in CADPS2. This condensed plot does not display SNPs in the region that are not ADT hits. The plot reveals that the first two SNPs are in LD in HapMap CEU data, but they are not in LD with the third SNP.
(0.64 MB TIF)

Figure S5 (A) Chromosome 20: Condensed Haploview r2 LD Plot for the six ADT hits (rs1923342, rs6131447, rs6109753, rs6105047, rs13433297, rs6074591) proximal to ISM1. This condensed plot does not display SNPs in the region that are not ADT hits. Furthermore, the latter two SNPs (rs13433297, rs6074591) could not be plotted with the former four due to Haploview constraints. In any case, r2 values between the former four and latter two SNPs were negligible in HapMap CEU data. (B) Chromosome 20: Haploview r2 LD Plot for the four ADT hits (rs8120608, rs367114, rs453573, rs6043472) within MACROD2. The SNPs (rs8120608, rs6043472) could not be plotted with the other two due to Haploview constraints. In any case, r2 values between these two SNPs and the two plotted SNPs were negligible. The plot indicates that the two plotted SNPs are in LD in HapMap CEU data, as highlighted by the yellow circle. (C) Chromosome 20: Condensed Haploview r2 LD Plot for the three ADT hits (rs6044739, rs6075193, rs6080665) within PCSK2. This condensed plot does not display SNPs in the region that are not ADT hits. The plot clearly indicates that the first two SNPs are in LD in HapMap CEU data, as any blank red blocks indicate r2  = 1.0 (100). The first two are in very weak LD with the third. (D) Chromosome 20: Condensed Haploview r2 LD Plot for the three ADT hits (rs4812744, rs6124601, rs6130470) proximal to TOX2. This condensed plot does not display SNPs in the region that are not ADT hits. The SNP rs4812744 could not be plotted with the other two due to Haploview constraints. The plot clearly indicates that the two plotted SNPs are in LD in HapMap CEU data.
(0.67 MB TIF)

Figure S6 Gene Ontology acyclic directed graph showing the most enriched terms and their ancestor terms. The significant terms are colored in green, with darker shading indicating greater statistical significance. The arrow colors indicate type of relationship between the GO categories, with black signifying “is a” and green signifying “regulates.”
(1.16 MB TIF)

Table S1 Top-scoring SNPs using ADT analysis (p≤0.005) from 178 TCGA samples.
(0.07 MB XLS)

## References

[1] Nowell PC (1976). The clonal evolution of tumor cell populations.. Science.

[2] de Koning JP, Wakabayashi Y, Nagase H, Mao JH, Balmain A (2007). Convergence of congenic mapping and allele-specific alterations in tumors for the resolution of the Skts1 skin tumor susceptibility locus.. Oncogene.

[3] Nagase H, Mao JH, Balmain A (2003). Allele-specific Hras mutations and genetic alterations at tumor susceptibility loci in skin carcinomas from interspecific hybrid mice.. Cancer Res.

[4] Ewart-Toland A, Briassouli P, de Koning JP, Mao JH, Yuan J (2003). Identification of Stk6/STK15 as a candidate low-penetrance tumor-susceptibility gene in mouse and human.. Nat Genet.

[5] Hienonen T, Salovaara R, Mecklin JP, Jarvinen H, Karhu A (2006). Preferential amplification of AURKA 91A (Ile31) in familial colorectal cancers.. Int J Cancer.

[6] Tuupanen S, Niittymaki I, Nousiainen K, Vanharanta S, Mecklin JP (2008). Allelic imbalance at rs6983267 suggests selection of the risk allele in somatic colorectal tumor evolution.. Cancer Res.

[7] Olcaydu D, Harutyunyan A, Jager R, Berg T, Gisslinger B (2009). A common JAK2 haplotype confers susceptibility to myeloproliferative neoplasms.. Nat Genet.

[8] Kilpivaara O, Mukherjee S, Schram AM, Wadleigh M, Mullally A (2009). A germline JAK2 SNP is associated with predisposition to the development of JAK2(V617F)-positive myeloproliferative neoplasms.. Nat Genet.

[9] Jones AV, Chase A, Silver RT, Oscier D, Zoi K (2009). JAK2 haplotype is a major risk factor for the development of myeloproliferative neoplasms.. Nat Genet.

[10] Dewal N, Freedman ML, LaFramboise T, Pe'er I (2010). Power to detect selective allelic amplification in genome-wide scans of tumor data.. Bioinformatics.

[11] Spielman RS, McGinnis RE, Ewens WJ (1993). Transmission test for linkage disequilibrium: the insulin gene region and insulin-dependent diabetes mellitus (IDDM).. Am J Hum Genet.

[12] LaFramboise T, Weir BA, Zhao X, Beroukhim R, Li C (2005). Allele-specific amplification in cancer revealed by SNP array analysis.. PLoS Comput Biol.

[13] (2008). Comprehensive genomic characterization defines human glioblastoma genes and core pathways.. Nature.

[14] Ashburner M, Ball CA, Blake JA, Botstein D, Butler H (2000). Gene ontology: tool for the unification of biology. The Gene Ontology Consortium.. Nat Genet.

[15] Wrensch M, Jenkins RB, Chang JS, Yeh RF, Xiao Y (2009). Variants in the CDKN2B and RTEL1 regions are associated with high-grade glioma susceptibility.. Nat Genet.

[16] Price AL, Patterson NJ, Plenge RM, Weinblatt ME, Shadick NA (2006). Principal components analysis corrects for stratification in genome-wide association studies.. Nat Genet.

[17] Gomes AL, Reis-Filho JS, Lopes JM, Martinho O, Lambros MB (2007). Molecular alterations of KIT oncogene in gliomas.. Cell Oncol.

[18] Holtkamp N, Ziegenhagen N, Malzer E, Hartmann C, Giese A (2007). Characterization of the amplicon on chromosomal segment 4q12 in glioblastoma multiforme.. Neuro Oncol.

[19] Sihto H, Tynninen O, Butzow R, Saarialho-Kere U, Joensuu H (2007). Endothelial cell KIT expression in human tumours.. J Pathol.

[20] Reardon DA, Dresemann G, Taillibert S, Campone M, van den Bent M (2009). Multicentre phase II studies evaluating imatinib plus hydroxyurea in patients with progressive glioblastoma.. Br J Cancer.

[21] Razis E, Selviaridis P, Labropoulos S, Norris JL, Zhu MJ (2009). Phase II study of neoadjuvant imatinib in glioblastoma: evaluation of clinical and molecular effects of the treatment.. Clin Cancer Res.

[22] Verhaak RG, Hoadley KA, Purdom E, Wang V, Qi Y (2010). Integrated genomic analysis identifies clinically relevant subtypes of glioblastoma characterized by abnormalities in PDGFRA, IDH1, EGFR, and NF1.. Cancer Cell.

[23] Yajnik V, Paulding C, Sordella R, McClatchey AI, Saito M (2003). DOCK4, a GTPase activator, is disrupted during tumorigenesis.. Cell.

[24] Heasman SJ, Ridley AJ (2008). Mammalian Rho GTPases: new insights into their functions from in vivo studies.. Nat Rev Mol Cell Biol.

[25] Ueda S, Fujimoto S, Hiramoto K, Negishi M, Katoh H (2008). Dock4 regulates dendritic development in hippocampal neurons.. J Neurosci Res.

[26] Jarzynka MJ, Hu B, Hui KM, Bar-Joseph I, Gu W (2007). ELMO1 and Dock180, a bipartite Rac1 guanine nucleotide exchange factor, promote human glioma cell invasion.. Cancer Res.

[27] Huang PH, Xu AM, White FM (2009). Oncogenic EGFR signaling networks in glioma.. Sci Signal.

[28] Ekstrand AJ, Sugawa N, James CD, Collins VP (1992). Amplified and rearranged epidermal growth factor receptor genes in human glioblastomas reveal deletions of sequences encoding portions of the N- and/or C-terminal tails.. Proc Natl Acad Sci U S A.

[29] Wong AJ, Bigner SH, Bigner DD, Kinzler KW, Hamilton SR (1987). Increased expression of the epidermal growth factor receptor gene in malignant gliomas is invariably associated with gene amplification.. Proc Natl Acad Sci U S A.

[30] Brandes AA, Franceschi E, Tosoni A, Hegi ME, Stupp R (2008). Epidermal growth factor receptor inhibitors in neuro-oncology: hopes and disappointments.. Clin Cancer Res.

[31] Mellinghoff IK, Wang MY, Vivanco I, Haas-Kogan DA, Zhu S (2005). Molecular determinants of the response of glioblastomas to EGFR kinase inhibitors.. N Engl J Med.

[32] Ohgaki H, Kleihues P (2007). Genetic pathways to primary and secondary glioblastoma.. Am J Pathol.

[33] Alimonti A, Carracedo A, Clohessy JG, Trotman LC, Nardella C Subtle variations in Pten dose determine cancer susceptibility.. Nat Genet.

[34] Knudson AG (1971). Mutation and cancer: statistical study of retinoblastoma.. Proc Natl Acad Sci U S A.

[35] Dumont P, Leu JI, Della Pietra AC, George DL, Murphy M (2003). The codon 72 polymorphic variants of p53 have markedly different apoptotic potential.. Nat Genet.

[36] To MD, Wong CE, Karnezis AN, Del Rosario R, Di Lauro R (2008). Kras regulatory elements and exon 4A determine mutation specificity in lung cancer.. Nat Genet.

[37] Bauer S, Grossmann S, Vingron M, Robinson PN (2008). Ontologizer 2.0—a multifunctional tool for GO term enrichment analysis and data exploration.. Bioinformatics.

