# Supplementary figures and images for: Allelic Selection of Amplicons in Glioblastoma Revealed by Combining Somatic and Germline Analysis
Source: PLoS Genet. 2010 Sep 2;6(9):e1001086. doi: 10.1371/journal.pgen.1001086 (PMC2932683; doi:10.1371/journal.pgen.1001086)

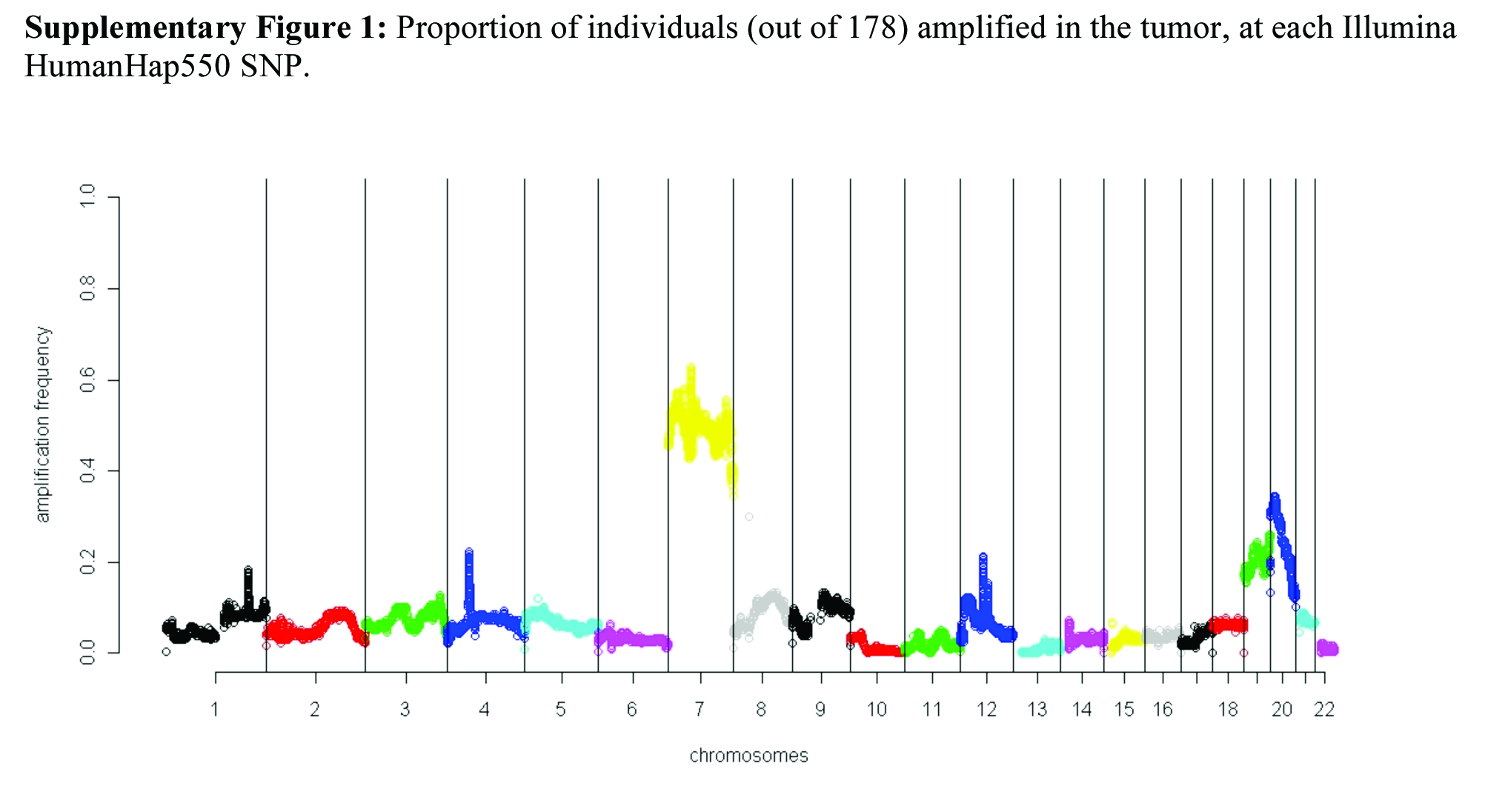

Supplement: Figure S1 — Proportion of individuals (out of 178) amplified in the tumor, at each Illumina HumanHap550 SNP. (1.01 MB TIF) [file pgen.1001086.s001.tif]

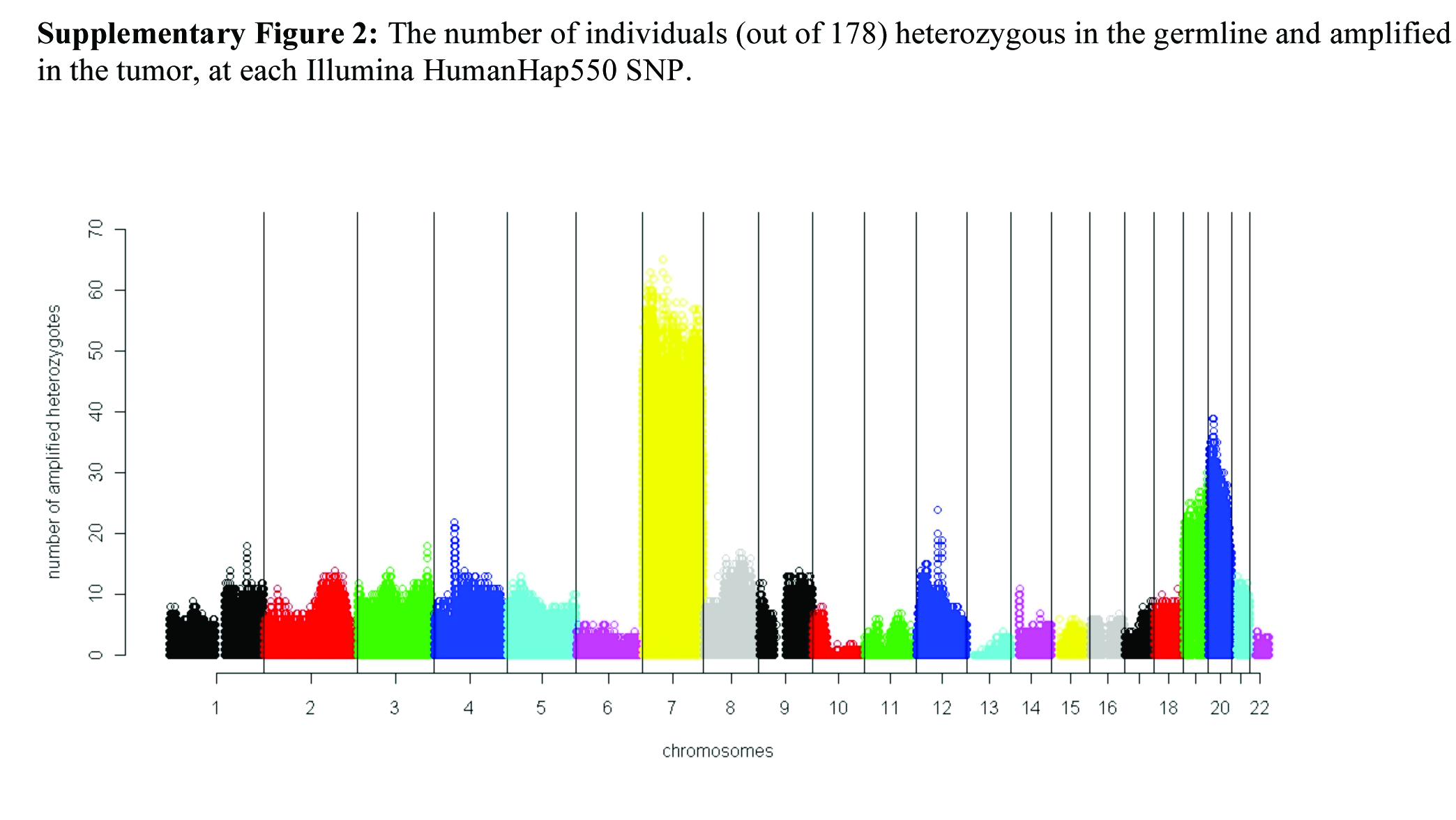

Supplement: Figure S2 — The number of individuals (out of 178) heterozygous in the germline and amplified in the tumor, at each Illumina HumanHap550 SNP. (1.13 MB TIF) [file pgen.1001086.s002.tif]

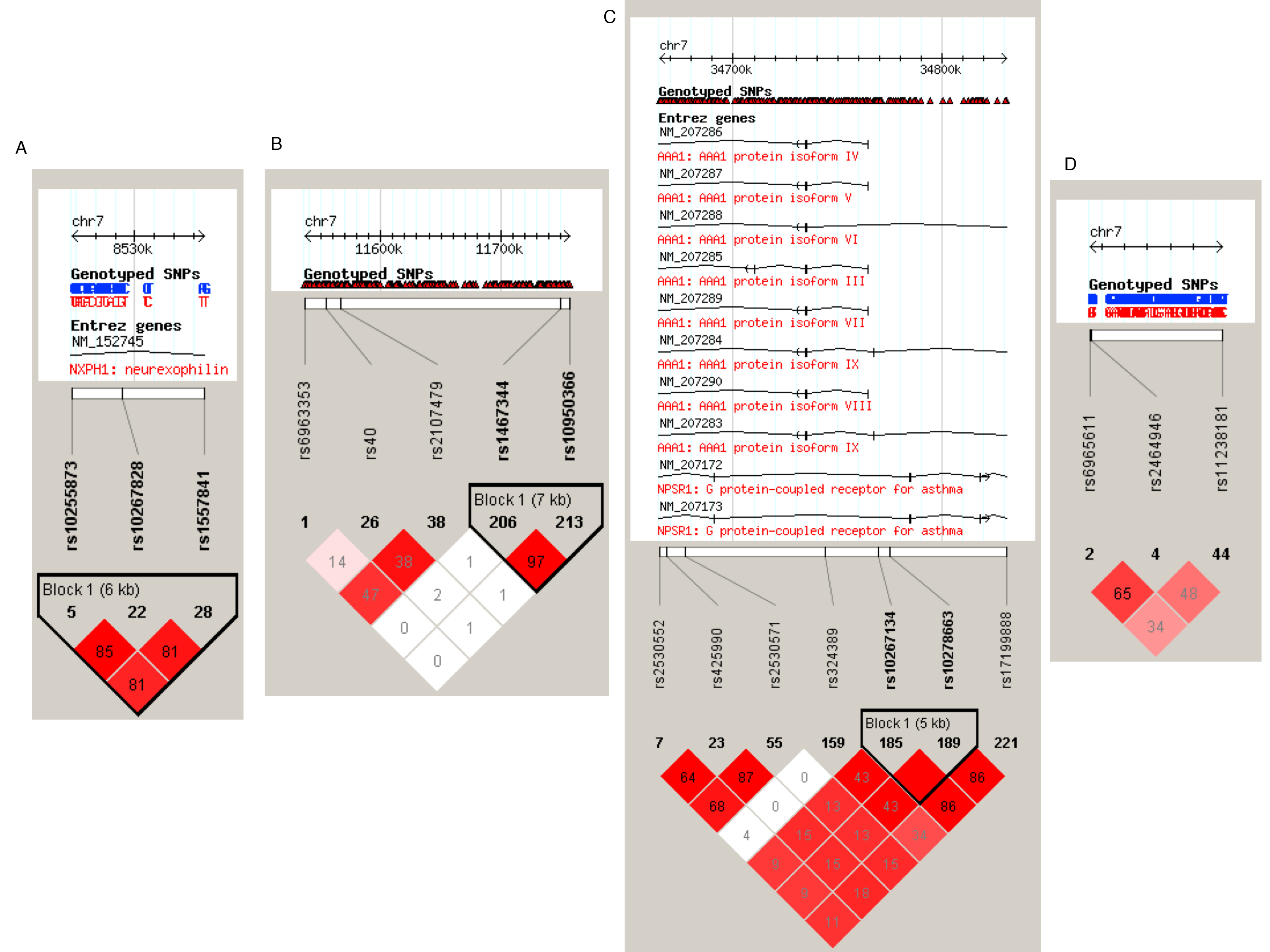

Supplement: Figure S3 — (A) Chromosome 7: Condensed Haploview r2 LD Plot for the three ADT hits (rs10255873, rs10267828, rs1557841) in NXPH1. This condensed plot does not display SNPs in the region that are not ADT hits. The plot indicates that the SNPs are in LD in HapMap CEU data. (B) Chromosome 7: Condensed Haploview r2 LD Plot for the five ADT hits (rs6963353, rs40, rs2107479, rs1467344, rs10950366) in THSD7A. This condensed plot does not display SNPs in the region that are not ADT hits. The plot indicates that the latter two SNPs are in LD in HapMap CEU data. (C) Chromosome 7: Condensed Haploview r2 LD Plot for the seven ADT (rs2530552, rs425990, rs2530571, rs324389, rs10267134, rs10278663, rs17199888) in NPSR1. This condensed plot does not display SNPs in the region that are not ADT hits. The plot indicates that the first three SNPs are in reasonable LD in HapMap CEU data, and the same holds for the latter three SNPs. Any blank red blocks indicate r2 = 1.0 (100). (D) Chromosome 7: Condensed Haploview r2 LD Plot for the three ADT hits (rs6965611, rs2464946, rs11238181) in a region with no known gene. This condensed plot does not display SNPs in the region that are not ADT hits. The plot indicates that the first two SNPs exhibit some LD in the HapMap CEU data. (1.09 MB TIF) [file pgen.1001086.s003.tif]

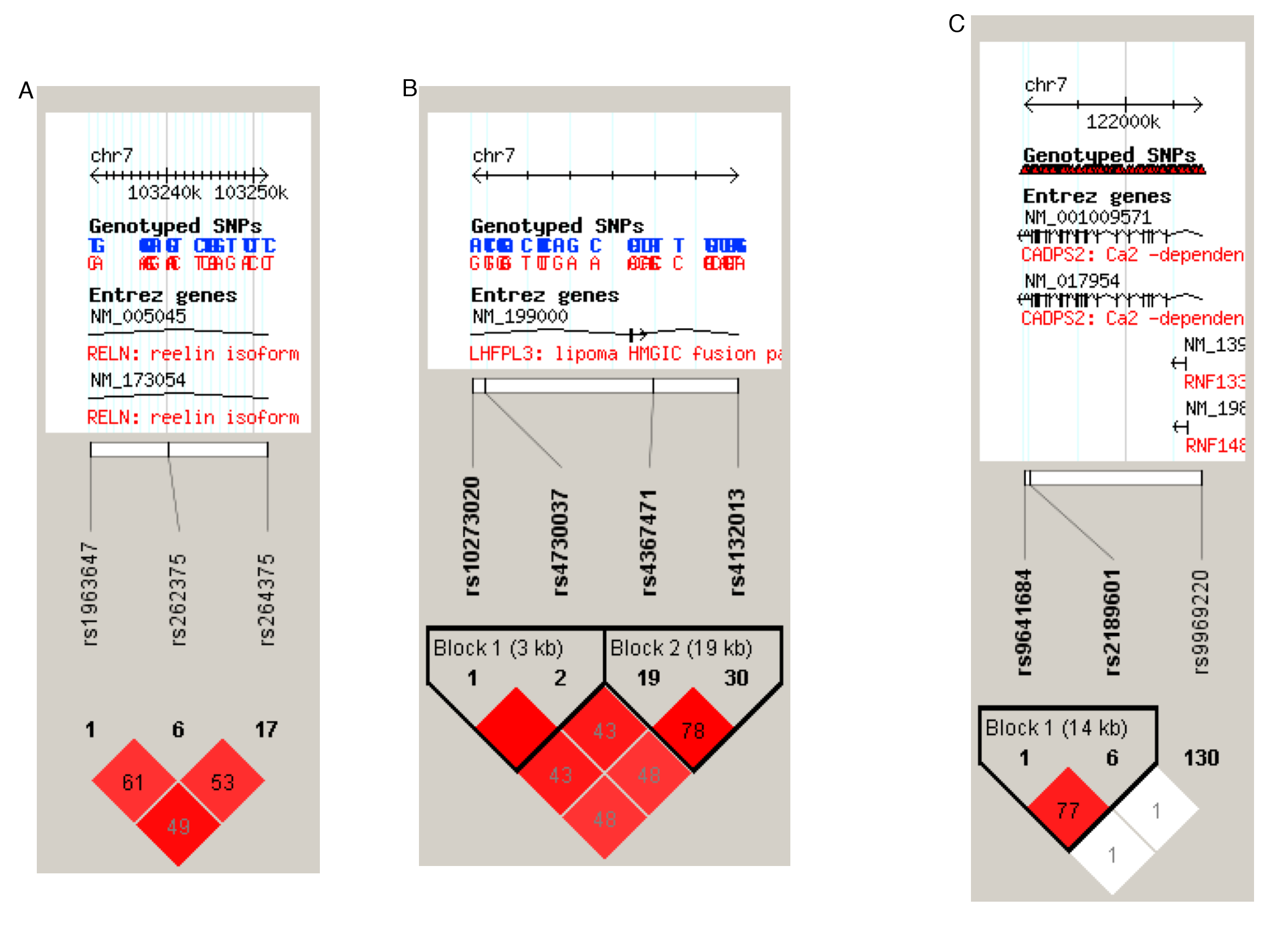

Supplement: Figure S4 — (A) Chromosome 7: Condensed Haploview r2 LD Plot for the three ADT hits (rs1963647, rs262375, rs264375) in RELN. This condensed plot does not display SNPs in the region that are not ADT hits. The plot indicates that the SNPs display some level of LD in HapMap CEU data. (B) Chromosome 7: Condensed Haploview r2 LD Plot for the four ADT hits (rs10273020, rs4730037, rs4367471, rs4132013) in LHFPL3. This condensed plot does not display SNPs in the region that are not ADT hits. The plot reveals that the first two SNPs are in strong LD in HapMap CEU data, as blank red blocks indicate r2 = 1.0 (100). The latter two SNPs also show LD in HapMap CEU data. (C) Chromosome 7: Condensed Haploview r2 LD Plot for the three ADT hits (rs9641684, rs2189601, rs9969220) in CADPS2. This condensed plot does not display SNPs in the region that are not ADT hits. The plot reveals that the first two SNPs are in LD in HapMap CEU data, but they are not in LD with the third SNP. (0.64 MB TIF) [file pgen.1001086.s004.tif]

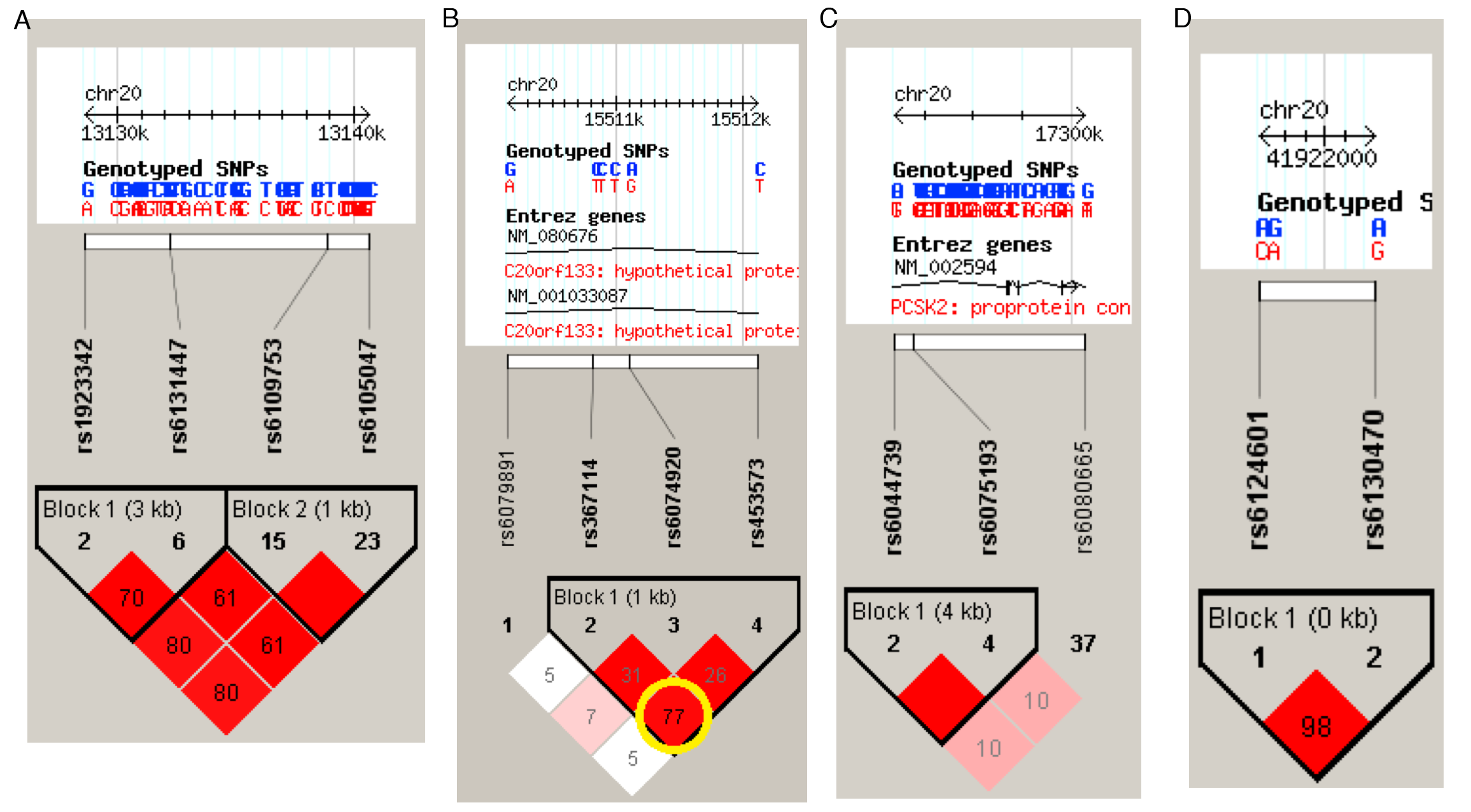

Supplement: Figure S5 — (A) Chromosome 20: Condensed Haploview r2 LD Plot for the six ADT hits (rs1923342, rs6131447, rs6109753, rs6105047, rs13433297, rs6074591) proximal to ISM1. This condensed plot does not display SNPs in the region that are not ADT hits. Furthermore, the latter two SNPs (rs13433297, rs6074591) could not be plotted with the former four due to Haploview constraints. In any case, r2 values between the former four and latter two SNPs were negligible in HapMap CEU data. (B) Chromosome 20: Haploview r2 LD Plot for the four ADT hits (rs8120608, rs367114, rs453573, rs6043472) within MACROD2. The SNPs (rs8120608, rs6043472) could not be plotted with the other two due to Haploview constraints. In any case, r2 values between these two SNPs and the two plotted SNPs were negligible. The plot indicates that the two plotted SNPs are in LD in HapMap CEU data, as highlighted by the yellow circle. (C) Chromosome 20: Condensed Haploview r2 LD Plot for the three ADT hits (rs6044739, rs6075193, rs6080665) within PCSK2. This condensed plot does not display SNPs in the region that are not ADT hits. The plot clearly indicates that the first two SNPs are in LD in HapMap CEU data, as any blank red blocks indicate r2 = 1.0 (100). The first two are in very weak LD with the third. (D) Chromosome 20: Condensed Haploview r2 LD Plot for the three ADT hits (rs4812744, rs6124601, rs6130470) proximal to TOX2. This condensed plot does not display SNPs in the region that are not ADT hits. The SNP rs4812744 could not be plotted with the other two due to Haploview constraints. The plot clearly indicates that the two plotted SNPs are in LD in HapMap CEU data. (0.67 MB TIF) [file pgen.1001086.s005.tif]

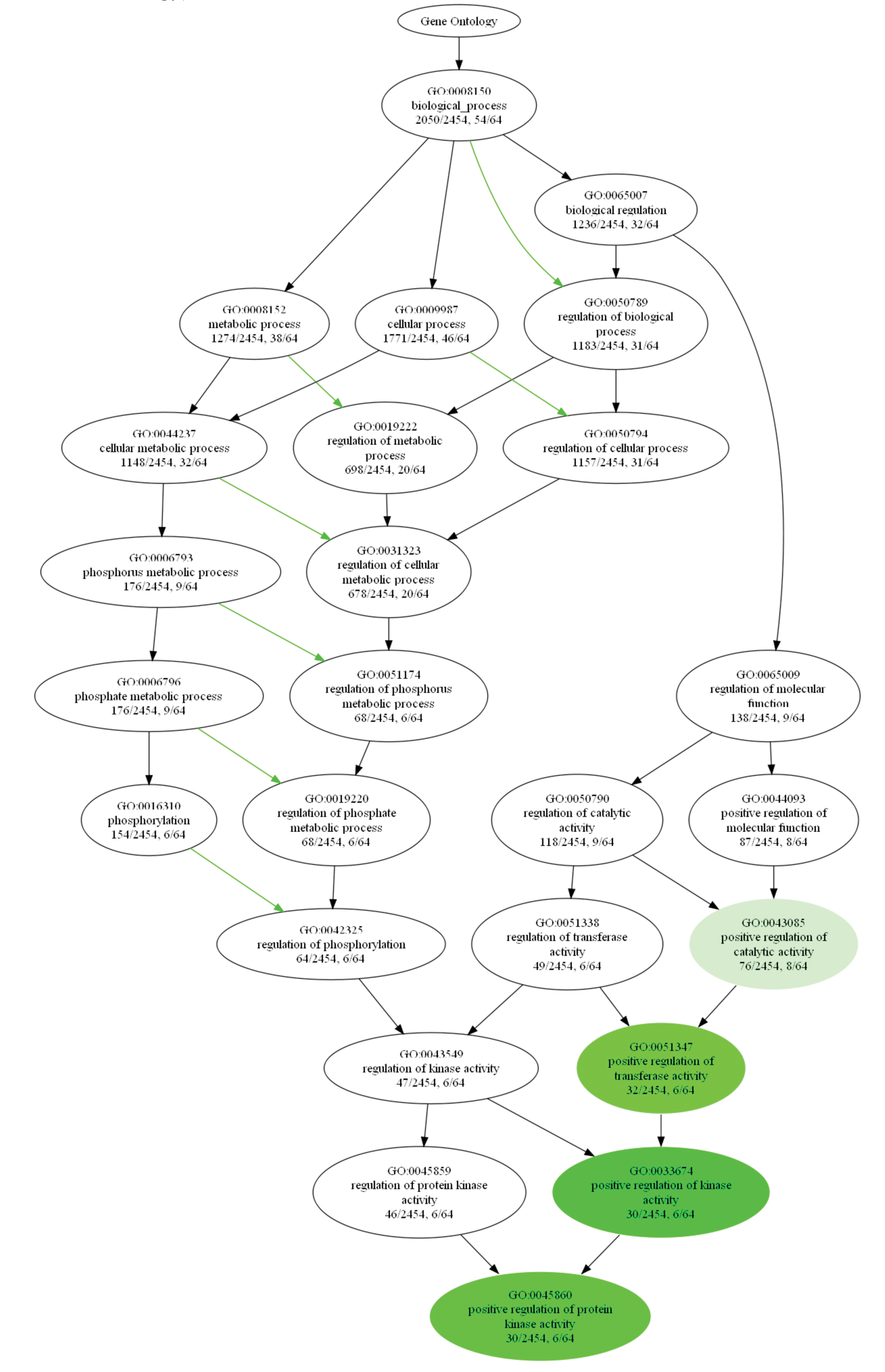

Supplement: Figure S6 — Gene Ontology acyclic directed graph showing the most enriched terms and their ancestor terms. The significant terms are colored in green, with darker shading indicating greater statistical significance. The arrow colors indicate type of relationship between the GO categories, with black signifying “is a” and green signifying “regulates.” (1.16 MB TIF) [file pgen.1001086.s006.tif]
